# Association of Plasma Aflatoxin With Persistent Detection of Oncogenic Human Papillomaviruses in Cervical Samples From Kenyan Women Enrolled in a Longitudinal Study

**DOI:** 10.21203/rs.3.rs-2468599/v1

**Published:** 2023-01-27

**Authors:** Yan Tong, Philip Tonui, Omenge Orang’o, Jianjun Zhang, Titus Maina, Kapten Muthoka, John Groopman, Joshua Smith, Erin Madeen, Aaron Ermel, Patrick Loehrer, Darron Brown

**Affiliations:** Indiana University School of Medicine; Moi University; Moi University; Indiana University; Maseno University; Academic Model Providing Access to Healthcare (AMPATH); Johns Hopkins Bloomberg School of Public Health; Johns Hopkins Bloomberg School of Public Health; Johns Hopkins Bloomberg School of Public Health; Indiana University School of Medicine; Indiana University Melvin and Bren Simon Comprehensive Cancer Center; Indiana University School of Medicine

## Abstract

**Background:**

Cervical cancer is common among Kenyan women and is caused by oncogenic human papillomaviruses (HR-HPV). Identification of factors that increase HR-HPV persistence is critically important. Kenyan women exposed to aflatoxin have an increased risk of cervical HR-HPV detection. This analysis was performed to examine associations between aflatoxin and HR-HPV persistence.

**Methods:**

Kenyan women were enrolled in a prospective study. The analytical cohort for this analysis included 67 HIV-uninfected women (mean age 34 years) who completed at least two of three annual study visits and had an available blood sample. Plasma aflatoxin was detected using ultra-high pressure liquid chromatography (UHPLC)-isotope dilution mass spectrometry. Annual cervical swabs were tested for HPV (Roche Linear Array). Ordinal logistic regression models were tted to examine associations of aflatoxin and HPV persistence.

**Results:**

Aflatoxin was detected in 59.7% of women and was associated with higher risk of persistent detection of any HPV type (OR = 3.03, 95%CI = 1.08–8.55, P = 0.036), HR-HPV types (OR = 3.63, 95%CI = 1.30–10.13, P = 0.014), and HR-HPV types not included in the 9-valent HPV vaccine (OR = 4.46, 95%CI = 1.13–17.58, P = 0.032).

**Conclusions:**

Aflatoxin detection was associated with increased risk of HR-HPV persistence in Kenyan women. Further studies are needed to determine if aflatoxin synergistically interacts with HR-HPV to increase cervical cancer risk.

## Introduction

Cervical cancer is a common malignancy among Kenyan women [1–3]. The incidence rate of cervical cancer in Kenya is 31.3 per 100,000 women per year and the mortality rate is 25 per 100,000 women per year, figures considerably higher than those in wealthy countries [4, 5]. Oncogenic types of human papillomaviruses (“high-risk”, or HR-HPV) are the causative agents of cervical cancer. However, only a small percentage of women infected with HR-HPV will develop cancer, indicating the importance of cofactors associated with HR-HPV persistence that contribute to the occurrence of cervical cancer [6]. Women with persistent infection with oncogenic HPV are at significantly higher risk of cervical cancer [7]. HIV infection is one cofactor that imparts a higher likelihood of HR-HPV persistence [2, 8, 9]. HR-HPV detection and persistence are also prevalent among Kenyan women who are not HIV-infected [10, 11].

Dietary aflatoxin may be another risk factor for HR-HPV persistence that is additive with HIV infection. Aflatoxin is a potent carcinogen and immunosuppressive agent produced by certain strains of Aspergillus, a mold that infects corn crops [12–14]. Large percentages of people living in sub-Saharan African countries are exposed to aflatoxin. We previously showed in a cross-sectional analysis that plasma aflatoxin biomarkers were detected among 57% of HIV-uninfected Kenyan women enrolled in a prospective study of HPV epidemiology and associated with cervical detection of A9 HPV types [15]. An additional analysis was performed using longitudinal data from this cohort to examine associations between plasma aflatoxin detection and cervical HR-HPV persistence.

## Methods

### Study Population

Kenyan women were enrolled from September 2015 to October 2016 at the Academic Model Providing Access to Healthcare (AMPATH) Cervical Cancer Screening Program (CCSP) at Moi Teaching and Referral Hospital in Eldoret, Kenya [16]. They were participants in a prospective cohort study investigating biological, behavioral, and environmental risk factors for oncogenic HPV persistence, a part of the East African Consortium for Human Papillomavirus and Cervical Cancer in Women Living with HIV/AIDS [16]. Details of study enrollment procedures have been previously published [11]. Briefly, women aged 18 to 60 years living within 30 km of Eldoret presenting for screening at the CCSP were asked to participate in the study if they had a normal visual inspection with acetic acid (VIA) of the uterine cervix that day.

A total of 223 women consented to participation and enrolled in the study, including 116 HIV-infected and 107 HIV-uninfected women. Plasma obtained at enrollment was available for 87 of 107 HIV-uninfected women, but no plasma sample was available for the HIV-infected women. Of the 87 HIV-uninfected women with available plasma, 67 women had at least two adequate cervical swab samples (based on beta globin testing) obtained at enrollment and at 12- or/and 24-month follow-up visits. These 67 women represented the analytical cohort for this post-hoc analysis.

### Interview and questionnaire

Structured face-to-face interviews of the participants by trained researchers were conducted at enrollment to capture social, behavioral, and biological information, including age, marital status, educational level, home ownership, walking distance to the local clinic, number of lifetime sexual partners, and age of first sex [11].

### Cervical swab and plasma sample collection

A cervical swab for HPV testing was collected by a nurse or physician as part of the inspection for cervical cancer screening. Swabs were placed in standard transport media and frozen at −80°C in the AMPATH Reference Laboratory. Plasma was collected and frozen at −20°C at the same laboratory.

### HPV testing

Cervical specimens were transported on dry ice to the Kenya Medical Research Institute-University of Massachusetts Medical School (KEMRI-UMMS) Laboratory for processing, DNA extraction, and subsequent genotyping [11]. The Roche Linear Array was used to determine HPV types (Roche Molecular Systems, Inc., Branchburg, NJ USA) as previously described [17]. HPV 16-positive, negative, and human beta-globin (used to assess specimen adequacy) controls provided by the manufacturer were tested with each batch of samples.

HPV types were grouped into “high-risk” (HR-HPV) and “low-risk” (LR-HPV) based on the designation in the Roche Linear Array instructions, or HR-HPV types as designated by the International Agency for the Research on Cancer (IARC) [18]. HPV types were further grouped into A9 and A7 types [19]. The specific HPV types included in each group are detailed in Results.

### Aflatoxin-albumin adduct (AFB_1_-lys) detection in plasma samples

Plasma aflatoxin B1-lysine (AFB_1_-lys) was measured at the Department of Environmental Health and Engineering of the Johns Hopkins Bloomberg School of Public Health, using a minor variation of the method reported by McCoy and colleagues [15, 20]. Briefly, plasma (150 μL) was spiked with an internal standard (0.5 ng AFB_1_-d4-lysine in 100 μL), combined with Pronase (EMD Millipore, Billerica MA, USA) protease solution (3.25 mg in 0.5 mL phosphate-buffered saline), and incubated for 18 hours at 37°C. Solid-phase extraction–processed samples (Oasis MAX columns; Waters, Milford, MA, USA) were analyzed with ultra-high pressure liquid chromatography (UHPLC)-isotope dilution mass spectrometry on a ThermoFisher Scientific (San Jose, CA, USA) system composed of a Vanquish UHPLC and a TSQ Quantis triple quadrupole mass spectrometer in positive electrospray ionization mode [21, 22].

### Persistent HPV detection

Type-specific HPV testing results obtained from the enrollment, 12-month and 24-month cervical samples were combined to determine the detection category of each specific HPV type for each woman. Three categories of HPV detection were determined: No detection, Incident Detection, and Persistent Detection. To be included, two or three of a participant’s cervical samples (Enrollment, 12-month, or 24-month) had to be available; one of the three samples could be missing. The type-specific HPV detection categories were defined as follows: I. No detection: No detection for the specific HPV type at any of the three time-points; II. Incident detection: One sample positive for detection of a specific HPV type, but other samples were negative for that type; III. Persistent detection: Two samples taken one year apart, or two years apart were positive for detection of a specific HPV type. The third sample could be negative for that type (or missing).

At the level of study participants, a woman’s HPV detection status was defined as the highest level of HPV detection category in the descending order of “Persistent detection,” “Incident detection,” and “No detection” among her type-specific HPV detection episodes within a combined group of specific HPV types. For example, a woman is classified as at the status of persistent detection in HR-HPV if any type-specific “persistence detection” episode is identified among the HR-HPV types. The subsequent analysis of HPV detection was conducted at the level of participant.

### Statistical analysis

Demographic and behavioral characteristics of participants at enrollment (age, marital status, educational level, home ownership, walking distance to health care of ≥60 min, number of lifetime sex partners, and age of first sex) were summarized by descriptive statistics and compared between women with and without detectable plasma AFB_1_-lys using t-tests, chi-square tests, or Wilcoxon rank sum tests. Frequencies and percentages of HPV detections (“No detection”, “Incident detection” and “Persistent detection”) in women were compared between those with and without detectable plasma AFB_1_-lys using chi-square tests or Fisher’s exact tests. Plasma AFB1-lys concentration (pg/uL) were summarized in mean, standard deviation (std), median and interquartile range (IQR) and compared among women with different HPV detection status using Wilcoxon rank sum tests. In addition, ordinal logistic regression models were fitted to examine associations of HPV detection (persistent detection vs. incident detection vs. no detection) with plasma aflatoxin detection, controlling for demographic and behavioral characteristics of the women as confounders. The proportional odds assumption was examined for each fitted ordinal logistic regression model to ensure validity of the model. All analyses were performed using SAS Version 9.4 (Cary, NC).

### Ethics considerations

## Results

### Overall characteristics of participants and aflatoxin (AFB_1_-lys) detection

The median age (IQR) at enrollment of 67 participants with an available plasma sample and valid HPV testing results was 34.0 (30.0, 38.0) years (range 21 to 46 years) (Table 1). Of 67 women, 27 (40.3%) had no detection of AFB_1_-lys in plasma, and 40 women (59.7%) had AFB_1_-lys detected (Table 1). Women with and without detectable plasma AFB_1_-lys were not significantly different in age, being married, having more than secondary school education, home ownership, living at a walking distance to health care of ≥60 minutes, number of lifetime sex partners, or age of first sex (Table 1).

A total of 87 women in the original cohort had plasma samples tested for AFB_1_-lys, including 67 women consisting of the analytical cohort and 20 women who did not complete at least two study visits. Comparisons between the 67 women in this analytical cohort and the 20 women who did not complete at least two visits were conducted with respect to the demographics/behavioral characteristics and plasma AFB1-lys detection/concentration. No significant differences in these variables were found between the two groups of women (data not shown).

### Association of plasma AFB_1_-lys detection with persistent HPV detection

Frequencies and percentages of HPV detections (“no detection”, “incident detection” and “persistent detection”) in women with and without plasma AFB_1_-lys detection and mean (STD) and median (IQR) of plasma AFB_1_-lys concentration (pg/uL) among women with different HPV detections are shown in Table 2. There was a trend of significantly increasing plasma AFB_1_-lys concentrations among women who had no detection, incident detection, or persistent detection for any HPV type (p = 0.036), HR-HPV types (p = 0.020), or vaccine-unprotected HR-HPV types (p = 0.017) (Table 2). Similar trends in increasing plasma AFB_1_-lys concentrations were observed for some other groups of HPV types, however, these were not significant (Table 2).

In addition, compared with women without detectable plasma AFB_1_-lys, women with detectable plasma AFB_1_-lys demonstrated significantly higher percentages of detection for any HPV type (22.5% vs. 11.1% for persistent detection and 55.0% vs. 37.0% for incident detection, p = 0.043) and HR-HPV type (22.5% vs. 7.4% for persistent detection and 45.0% vs. 29.6% for incident detection, p = 0.038). Similar patterns of HPV detections between women with and without detectable plasma AFB_1_-lys were found for all other HPV combination types except for LR-HPV types. However these were not statistically significant, possibly due to small sample sizes.

A total of 13 episodes of type-specific persistent HPV detections occurred in 12 women (Table 3). Among these episodes, 12 were HR-HPV types, including 10 episodes that occurred in 9 of 40 women with detectable plasma AFB_1_-lys, and 2 episodes occurring in 2 of 27 women without detectable plasma AFB_1_-lys (Table 3). HPV 18 was the most frequently detected persistent type (3 episodes), all occurring in women with detectable plasma AFB_1_-lys (Table 3).

Ordinal logistic regression analysis revealed that detectable plasma AFB_1_-lys was associated with a higher risk of persistent detection for any HPV type (OR = 3.03, 95%CI = 1.08–8.55, P = 0.036), HR-HPV types (OR = 3.63, 95%CI = 1.30–10.13, P = 0.014), and HR-HPV types not included in the 9-valent HPV vaccine (Vaccine-unprotected HR-HPV types) (OR = 4.46, 95%CI = 1.13–17.58, P = 0.032) after adjustment for established and suspected confounders (Table 4). The proportional odds assumption was validated for each ordinal logistic regression model. There was no statistically significant association of detectable plasma AFB_1_-lys with persistent detection of sub-groups of HR-HPV types, for HR-HPV types protected by the 9-valent HPV vaccine (Vaccine-protected HR-HPV types), or for low-risk (LR) HPV types (Supplemental Table 1).

## Discussion

In this longitudinal study, women with detectable aflatoxin biomarkers in plasma had a higher risk of persistent detection of oncogenic cervical HPV. Although only a small percentage of HPV-infected women will eventually develop cervical cancer, women with persistent detection of HR-HPV are at the highest risk for this malignancy [23, 24]. Aflatoxins are mycotoxins produced by certain Aspergillus species during growth or after harvesting of corn and several other crops [25]. These compounds are classified by the International Agency for Research on Cancer (IARC) as class I carcinogens [26]. In addition, aflatoxins are potent immunosuppressive agents [27–30]. Exposure to aflatoxins contributes heavily to the worldwide burden of hepatocellular carcinoma, but the contribution of aflatoxin exposure to other cancers is unknown [31, 32]. This study revealed an association between aflatoxin exposure and persistent HR-HPV detection, the major risk factor for cervical cancer.

A previous cross-sectional study showed significant associations between plasma aflatoxin biomarkers and detection of A9 HPV types in cervical samples among HIV-uninfected Kenyan women [15]. The current analysis employed a subset of the original cohort with the longitudinal follow-up data on HPV testing, disclosing the relationship of aflatoxins with persistent detections of HR-HPV, and raising the possibility that aflatoxin could be a contributing factor to cervical cancer. We are not aware of other studies describing an association of aflatoxin with HPV persistence, cervical dysplasia, or cancer.

It is possible that HR-HPV types and dietary aflatoxin act synergistically in increasing the risk of cervical cancer in Kenyan women. Aflatoxins have been detected in cervical tissue and could potentially act directly on cervical cells in the carcinogenic process, but this hypothesis has not been studied [33]. It is also possible the immunosuppression caused by aflatoxin could lead to poor immune control of oncogenic HPV infections, leading to persistence. These hypotheses need to be further investigated. In addition, it has a tremendous public health impact to investigate the role of the interaction between aflatoxin exposure and persistent HPV infection in the etiology, pathogenesis, and prevention of cervical cancer and its precursor lesions in large epidemiological studies especially in developing countries.

Aflatoxin exposure is widespread in many sub-Saharan African countries, largely due to consumption of contaminated corn, the major source of daily calories for many people, especially for poor families [34–36]. Leroy et al., showed higher serum aflatoxin levels from adult Kenyan women associated with lower household socio-economic status [37]. Women with the lowest socio-economic status also have the lowest rates of cervical cancer screening, and therefore bear the highest burden of cervical cancer [38, 39]. Aflatoxin, as a potential environmental risk factor of cervical cancer, demands more recognition for public health emphasis.

Some limitations of the present study include a modest sample size, as not all women initially analyzed for the association of aflatoxin detection and HR-HPV detection continued in the longitudinal study. However, our analysis showed that there were no significant differences in demographic/behavioral characteristics and plasma AFB_1_-lys detection/concentration between the women who remained in the original study and included in this analysis compared to those who did not continue in the original study. Another potential limitation is that dietary factors that modulate immune functions were not included as potential confounders in our data analysis, which could possibly distort the findings of the present study. For example, malnourishment may contribute to suppressed immunity and render such women more susceptible to the toxic effects of aflatoxin, and thus, more prone to persistent HPV infection [34, 40]. In addition, the results of our study may be subject to multiple comparisons due to a relatively large number of the models presented. However, this is unlikely because all exposure and outcome variables included in the constructed models were carefully selected in terms of the findings of previous studies and biological relevance.

In summary, detection of plasma aflatoxin biomarkers was associated with increased persistence of oncogenic HPV types, in cervical samples from HIV-uninfected Kenyan women. Further studies are needed to determine if exposure to aflatoxin interacts with HPV infection to modulate the risk of cervical cancer in Kenya and other developing countries. In addition, studies are underway to examine associations of aflatoxin exposure and HR-HPV infection on occurrence of cervical dysplasia in a cohort of HIV-infected sub-Saharan women, as HIV infection increases susceptibility to cervical cancer.

## Figures and Tables

**Table 1.** Demographic and behavioral characteristics of women with or without plasma AFB1-lys detection

Characteristics	Overall N=67	Plasma AFB1-lys Detection		
		No N=27	Yes N=40	P value
Median age in years (IQR)	34.0 (30.0, 38.0)	35.0 (30.0, 40.0)	33.5 (30.0, 38.0)	0.537^1^
Married	49 (73.1%)	19 (70.4%)	30 (75.0%)	0.675^2^
More than secondary school education	10 (14.9%)	2 (7.4%)	8 (20.0%)	0.185^3^
Home ownership	20 (29.9%)	7 (25.9%)	13 (32.5%)	0.564^2^
Walking distance to health care ≥60 mins	7 (10.4%)	2 (7.4%)	5 (12.5%)	0.693^3^
Median number of lifetime sex partners (IQR)	3.0 (1.0, 4.0)	2.0 (1.0, 4.0)	3.0 (1.0, 4.0)	0.588^4^
Median age of first sex (IQR)	18.0 (16.0, 20.0)	18.0 (17.0, 20.0)	18.0 (16.0, 20.0)	0.792^1^

1 P-value from t-test

2 P-value from Chi-square test

3 P-value from Fisher’s exact test

4 P-value from Wilcoxon rank sum test

**Table 2.** Frequency and percentage of HPV detection in 67 women with and without plasma AFB1-lys detection, and mean (standard deviation, or STD) and median (interquartile range, or IQR) of plasma AFB1-lys concentration (pg/uL).

HPV	HPV detection category	Plasma AFB1-lys concentration (pg/uL)			Plasma AFB1-lys detection		
		N, Mean (STD)	Median (IQR)	P-Value^13^	No (N=27)	Yes (N=40)	P-Value
					n (%)	n (%)	
Any HPV^1^	No Detection	23, 0.030 (0.042)	0.000 (0.0000.066)	0.036	14 (51.9)	9 (22.5)	0.043^11^
	Incident Detection	32, 0.050 (0.043)	0.052 (0.000–0.088)		10 (37.0)	22 (55.0)	
	Persistent Detection	12, 0.078 (0.068)	0.081 (0.014–0.098)		3 (11.1)	9 (22.5)	
HR-HPV^2^	No Detection	30, 0.034 (0.044)	0.000 (0.000–0.065)	0.020	17 (63.0)	13 (32.5)	0.038^11^
	Incident Detection	26, 0.049 (0.041)	0.050 (0.000–0.091)		8 (29.6)	18 (45.0)	
	Persistent Detection	11, 0.086 (0.066)	0.082 (0.027–0.098)		2 (7.4)	9 (22.5)	
IARC HR-HPV^3^	No Detection	35, 0.046 (0.057)	0.035 (0.000–0.073)	0.410	17 (63.0)	18 (45.0)	0.361^12^
	Incident Detection	24, 0.048 (0.042)	0.046 (0.000–0.093)		8 (29.6)	16 (40.0)	
	Persistent Detection	8, 0.058 (0.042)	0.078 (0.014–0.093)		2 (7.4)	6 (15.0)	
A9 HPV^4^	No Detection	45, 0.045 (0.053)	0.035 (0.000–0.073)	0.395	20 (74.1)	25 (62.5)	0.567^12^
	Incident Detection	17, 0.055 (0.043)	0.067 (0.000–0.094)		5 (18.5)	12 (30.0)	
	Persistent Detection	5, 0.054 (0.049)	0.082 (0.0000.089)		2 (7.4)	3 (7.5)	
Non-HPV 16 A9^5^	No Detection	48, 0.044 (0.052)	0.031 (0.000–0.073)	0.171	22 (81.5)	26 (65.0)	0.175^12^
	Incident Detection	15, 0.062 (0.041)	0.079 (0.023–0.095)		3 (11.1)	12 (30.0)	
	Persistent Detection	9, 0.047 (0.054)	0.045 (0.000–0.093)		2 (7.4)	2 (5.0)	
A7 HPV^6^	No Detection	55, 0.048 (0.050)	0.047 (0.000–0.085)	0.097	23 (85.2)	32 (80.0)	0.236^12^
	Incident Detection	8, 0.025 (0.029)	0.016 (0.000–0.050)		4 (14.8)	4 (10.0)	
	Persistent Detection	4, 0.096 (0.067)	0.086 (0.050–0.142)		0 (0.0)	4 (10.0)	
Non-HPV 18 A7^7^	No Detection	59, 0.049 (0.048)	0.047 (0.000–0.085)	0.090	23 (85.2)	36 (90.0)	0.661^12^
	Incident Detection	7, 0.024 (0.031)	0.000 (0.000–0.059)		4 (14.8)	3 (7.5)	
	Persistent Detection	1, 0.186 (−)	0.186 (0.186–0.186)		0 (0.0)	1 (2.5)	
Vaccine-protected HR-HPV^8^	No Detection	41, 0.046 (0.054)	0.040 (0.000–0.073)	0.505	18 (66.7)	23 (57.5)	0.625^12^
	Incident Detection	18, 0.047 (0.045)	0.042 (0.000–0.094)		7 (25.9)	11 (27.5)	
	Persistent Detection	8, 0.058 (0.042)	0.078 (0.014–0.093)		2 (7.4)	6 (15.0)	
Vaccine-unprotected HR-HPV^9^	No Detection	47, 0.038 (0.044)	0.023 (0.000–0.079)	0.017	23 (85.2)	24 (60.0)	0.064^12^
	Incident Detection	16, 0.054 (0.040)	0.063 (0.008–0.090)		4 (14.8)	12 (30.0)	
	Persistent Detection	4, 0.137 (0.071)	0.133 (0.077–0.198)		0 (0.0)	4 (10.0)	
LR-HPV^10^	No Detection	44, 0.045 (0.047)	0.038 (0.000–0.081)	0.414	19 (70.4)	25 (62.5)	0.284^12^
	Incident Detection	22, 0.057 (0.055)	0.058 (0.000–0.091)		7 (25.9)	15 (37.5)	
	Persistent Detection	1, 0.000 (−)	0.000 (0.000–0.000)		1 (3.7)	0 (0.0)	

1 Any HPV: HPV 6, 11, 16, 18, 26, 31, 33, 35, 39, 40, 42, 45, 51, 52, 53, 54, 55, 56, 58, 59, 61, 62, 64, 66, 67, 68, 70, 71, 72, 73, 81, 82, 83, 84, CP6108, IS39

2 HR-HPV (High-Risk HPV): HPV 16, 18, 26, 31, 33, 35, 39, 45, 51, 52, 53, 56, 58, 59, 66, 67, 68, 69, 70, 73, 82, IS39

3 ARC HR-HPV: HPV 16, 18, 31, 33, 35, 39, 45, 51, 52, 56, 58, 59, 66

4 A9 HPV: HPV 16, 31, 33, 35, 52, 58

5 Non-HPV 16 A9: HPV 31, 33, 35, 52, 58

6 A7 HPV: HPV 18, 39, 45, 59, 68

7 Non-HPV 18 A7: HPV 39, 45, 59, 68

8 Vaccine-protected HR-HPV: HPV 16, 18, 31, 33, 45, 52, 58

9 Vaccine-unprotected HR-HPV: HPV 26, 35, 39, 51, 53, 56, 59, 66, 67, 68, 69, 70, 73, 82, IS39

10 LR-HPV (Low-Risk HPV): HPV 6, 11, 40, 42, 54, 55, 61, 62, 64, 71, 72, 81, 83, 84, CP6108

11 P-value from Chi-square test

12 P-value from Fisher’s exact test

13 P-value from Wilcoxon rank sum test

**Table 3:** Episodes of type-specific persistent HPV detection and corresponding plasma AFB1-lys detection/concentration.

Subject ID	Type-specific HPV Detection					Plasma AFB1-lys	
	Type	Enrollment	12-month visit	24-month visit	Persistent detection	Detection	Concentration (pg/uL)
M028	HPV 16^2^	pos	missing	pos	2-year	yes	0.082
M060	HPV 18^2^	neg	pos	pos	1-year	yes	0.027
M120	HPV 18^2^	pos	pos	pos	2-year	yes	0.098
M189^1^	HPV 18^2^	neg	pos	pos	1-year	yes	0.073
M069	HPV 52^2^	neg	pos	pos	1-year	no	0.000
M121	HPV 52^2^	pos	pos	neg	1-year	yes	0.097
M168	HPV 53^3^	pos	pos	neg	1-year	yes	0.080
M066	HPV 58^2^	pos	pos	neg	1-year	no	0.000
M154	HPV 58^2^	neg	pos	pos	1-year	yes	0.089
M114	HPV 68^2^	neg	pos	pos	1-year	yes	0.186
M173	HPV 70^2^	pos	pos	neg	1-year	yes	0.209
M189^1^	HPV 70^2^	pos	pos	neg	1-year	yes	0.073
M155	HPV 83^2^	pos	pos	neg	1-year	no	0.000

1 M189 had two episodes of type-specific persistent HPV detections, one episode with HPV 18 and one episode with HPV 70

2 High-Risk HPV type

3 Low-Risk HPV type

**Table 4:** Ordinal logistic regression analyses of Any HPV, HR-HPV, Vaccine-protected HR-HPV, and Vaccine-unprotected HR-HPV detection (persistent detection vs. incidence detection vs. no detection) with plasma AFB1-lys detection and demographic/behavioral characteristics of women^5^.

Variables included in the model	Any HPV^1^		HR-HPV^2^		Vaccine-protected HR-HPV^3^		Vaccine-unprotected HR-HPV^4^	
	OR (95% CI)	P-value	OR (95% CI)	P-value	OR (95% CI)	P-value	OR (95% CI)	P-value
Plasma AFB1-lys detection	3.03 (1.088.55)	0.036	3.63 (1.3010.13)	0.014	1.55 (0.54–4.44)	0.413	4.46 (1.13–17.58)	0.032
Age	0.88 (0.79–0.98)	0.017	0.92 (0.83–1.02)	0.127	0.93 (0.83–1.03)	0.180	0.91 (0.79–1.05)	0.188
Married	0.28 (0.08–0.94)	0.039	0.63 (0.20–2.03)	0.442	0.38 (0.11–1.32)	0.128	0.58 (0.15–2.28)	0.437
More than secondary school education	1.13 (0.27–4.76)	0.868	0.72 (0.17–2.95)	0.645	1.41 (0.33–5.98)	0.643	1.10 (0.17–6.98)	0.918
Home ownership	2.43 (0.71–8.31)	0.156	1.60 (0.48–5.30)	0.441	1.45 (0.41–5.18)	0.563	1.78 (0.38–8.34)	0.465
Walking distance to health care ≥ 60 mins	1.46 (0.27–7.77)	0.657	0.83 (0.16–4.18)	0.820	0.46 (0.08–2.74)	0.397	2.27 (0.36–14.06)	0.380
Number of lifetime sex partners	1.07 (0.86–1.34)	0.544	1.10 (0.88–1.36)	0.405	1.02 (0.81–1.29)	0.880	0.99 (0.77–1.27)	0.918
Age of first sex	1.16 (0.98–1.38)	0.088	1.08 (0.92–1.28)	0.351	1.18 (0.99–1.40)	0.066	0.74 (0.57–0.97)	0.031

1 Any HPV: HPV 6, 11, 16, 18, 26, 31, 33, 35, 39, 40, 42, 45, 51, 52, 53, 54, 55, 56, 58, 59, 61, 62, 64, 66, 67, 68, 70, 71, 72, 73, 81, 82, 83, 84, CP6108, IS39

2 HR-HPV (High-Risk HPV): HPV 16, 18, 26, 31, 33, 35, 39, 45, 51, 52, 53, 56, 58, 59, 66, 67, 68, 69, 70, 73, 82, IS39

3 Vaccine-protected HR-HPV: HPV 16, 18, 31, 33, 45, 52, 58

4 Vaccine-unprotected HR-HPV: HPV 26, 35, 39, 51, 53, 56, 59, 66, 67, 68, 69, 70, 73, 82, IS39

## Data Availability

The data that support the findings of this study are available from the corresponding author (Dr. Brown and the additional authors) upon reasonable request and with permission of AMPATH.
